# Protist predation can favour cooperation within bacterial species

**DOI:** 10.1098/rsbl.2013.0548

**Published:** 2013-10-23

**Authors:** Ville-Petri Friman, Stephen P. Diggle, Angus Buckling

**Affiliations:** 1 Department of Zoology, University of Oxford, The Tinbergen Building, South Parks Road, Oxford OX1 3PS, UK; 2 Department of Biosciences, University of Exeter, Daphne du Maurier Building, Cornwall Campus, Penryn TR10 9EZ, UK; 3 School of Life Sciences, Centre for Biomolecular Sciences, University of Nottingham, University Park, Nottingham NG7 2RD, UK

**Keywords:** non-social selection, biofilm, pleiotropy, *Pseudomonas aeruginosa*, quorum sensing, *Tetrahymena pyriformis*

## Abstract

Here, we studied how protist predation affects cooperation in the opportunistic pathogen bacterium *Pseudomonas aeruginosa*, which uses quorum sensing (QS) cell-to-cell signalling to regulate the production of public goods. By competing wild-type bacteria with QS mutants (cheats), we show that a functioning QS system confers an elevated resistance to predation. Surprisingly, cheats were unable to exploit this resistance in the presence of cooperators, which suggests that resistance does not appear to result from activation of QS-regulated public goods. Instead, elevated resistance of wild-type bacteria was related to the ability to form more predation-resistant biofilms. This could be explained by the expression of QS-regulated resistance traits in densely populated biofilms and floating cell aggregations, or alternatively, by a pleiotropic cost of cheating where less resistant cheats are selectively removed from biofilms. These results show that trophic interactions among species can maintain cooperation within species, and have further implications for *P. aeruginosa* virulence in environmental reservoirs by potentially enriching the cooperative and highly infective strains with functional QS system.

## 1. Introduction

The costs and benefits of cooperation and cheating are dependent on environmental context [1–3]. Here, we investigate how a ubiquitous selection pressure, predation, affects selection for cooperation in the bacterium *Pseudomonas aeruginosa*, which cooperates by secreting and responding to quorum sensing (QS) signal molecules in the surrounding environment [4]. Once a threshold signal concentration is reached, bacteria switch on production of fitness-enhancing ‘public goods’. By regulating the production of public goods in this way, QS determines that they are released at high cell densities when they will be most beneficial [5]. Production of QS-dependent public goods can be exploited by cheats which do not produce the costly public goods because they do not respond to QS signal molecules [6].

Recent studies have reported a benefit of QS in the context of antipredator toxin production [7–9], and, where investigated, this toxin production can be exploited by social cheats [7]. However, in addition to regulating a large amount of secreted compounds that could act as public goods, transcriptomic and proteomic studies have also shown that QS regulates many intracellular metabolic functions [10], which could have direct fitness benefits to self (private goods), for example in terms of more efficient resource acquisition [2,5,10,11]. Given the massive effect of QS on gene regulation in *P. aeruginosa*, we investigated whether QS can confer direct or indirect fitness benefits against predation by culturing cooperating (PAO1) and QS-cheating (*lasI* and *lasR* mutants) *P. aeruginosa* bacterial strains in mono- and co-cultures, both in the absence and presence of a predatory protist, *Tetrahymena pyriformis*, in different resource concentrations. We show that a functioning QS system confers elevated resistance to predation that cheats are unable to exploit, probably through the production of more resistant cell aggregations and biofilms. Importantly, this elevated resistance does not appear to result from the activation of QS-regulated public goods. This suggests that antagonistic trophic interactions can indirectly favour QS-mediated cooperation, potentially leading to lower cheat frequency in natural microbial communities.

## 2. Material and methods

### (a) Study species

QS-positive, cooperating PAO1 and QS-defective, cheating *lasI* (signal-negative) and *lasR* (signal-blind) *P. aeruginosa* strains were used in this study [6]. The ‘signal-negative’ *lasI* mutant does not produce *N*-(3-oxododecanoyl)-L-homoserine lactone- (3O-C12-HSL) signal but still responds to signal, whereas the ‘signal-blind’ *lasR* mutant does not produce or respond to extracellular signal. Both types of mutants have been found in natural populations [6,11]. A single-celled Ciliate protist, *T. pyriformis* (CCAP 1630/1W) was used as a predator.

### (b) Mono- and co-culture experiments

Co-culture experiments were performed in 24-well plates (VWR). Bacteria were cultured alone and in 50 : 50 cooperator–cheat pairs (PAO1 versus *lasI* or *lasR*) in the absence and presence of protist in three different resource concentrations (1, 10 and 100% King's B (KB) medium: M9 salt solution supplemented with 10 g l^−1^ glycerol and 20 g l^−1^ proteose peptone). All wells were first inoculated with 1.5 ml of fresh KB medium after 5.1 × 10^5^ bacterial cells per strain were added to mono- and co-cultures, respectively. Half of the microcosms were inoculated with 60 cells of *T. pyriformis*. Cultures were propagated at 28°C in non-shaken conditions for 48 h before sampling. All populations were mixed throughout before estimating bacterial densities as colony forming units on KB plates after 24 h growth at 28°C. The cooperator–cheat co-cultures were also plated on gentamicin–KB plates (15 μg ml^−1^ of gentamicin) to estimate the proportion of gentamicin-resistant cheats. All treatments were replicated three times.

### (c) Resistance assays

Bacterial defence, that is susceptibility, was measured (i) as protist ability to reduce bacterial biofilm (growth as cell aggregations on surfaces) and (ii) as an increase in protist cell numbers after 24 h of co-cultivation (*N* = 4 in both cases). Overnight-grown bacterial strains were first diluted to even densities. Biofilm formation was measured by growing bacteria alone and in the presence of predator (inoculum of 60 protist cells) on 96-well plates at 28°C. After 24 h growth, protist densities were counted under microscope (10× magnification, Leica DM IL Led), and attached bacterial cells stained (50 µl of 1% crystal violet solution; VWR) and subsequently detached with 96% ethanol to measure the amount of biofilm with spectrophotometer (optical density at 600 nm). Bacterial toxicity to protist was measured as follows: overnight-grown bacterial monocultures were sterile filtered (0.22 µm millipore), and approximately 60 protist cells were inoculated to 200 µl of supernatant (including all exoenzymes; specific harmful compound not defined) to measure bacterial toxicity (protist cells counted after 24 h). Bacterial ability to form floating cell aggregations in the liquid phase of the culture media was visualized and imaged with Leica EC3 digital camera and Leica DM IL Led microscope (10× magnification).

### (d) Statistical analysis

Data were analysed with two-way ANOVA with focal species (bacterial strain), bacterial community composition, predation and resource concentration as categorical explanatory variables. Bacterial cell numbers were log transformed and proportional data arcsine transformed.

## 3. Results

Cooperative and cheat strains reached similar cell densities in monocultures regardless of the resource concentration (strain × resource: *F*_4,27_ = 0.4, *p* = 0.8, electronic supplementary material, figure S1*a*). Surprisingly, protists were driven to extinction in 10 and 100% resource concentrations by all bacterial strains during the co-culture experiments. According to separate resistance assays, bacterial supernatant was only toxic when it was derived from cooperator monocultures grown in 100% resource concentration (strain: *F*_3,11_ = 4.2, *p* = 0.03, electronic supplementary material, figure S1*b*). These results thus suggest that some other toxicity mechanism unrelated to QS signalling was also activated with both the cheats and cooperator when in direct contact with protist in intermediate and high resource concentrations (e.g. type three secretion system [9,12]). Thus, we concentrated on the effects of predation only within 1% resource concentration where protists were not driven extinct during the co-culture experiment.

**Figure 1.**
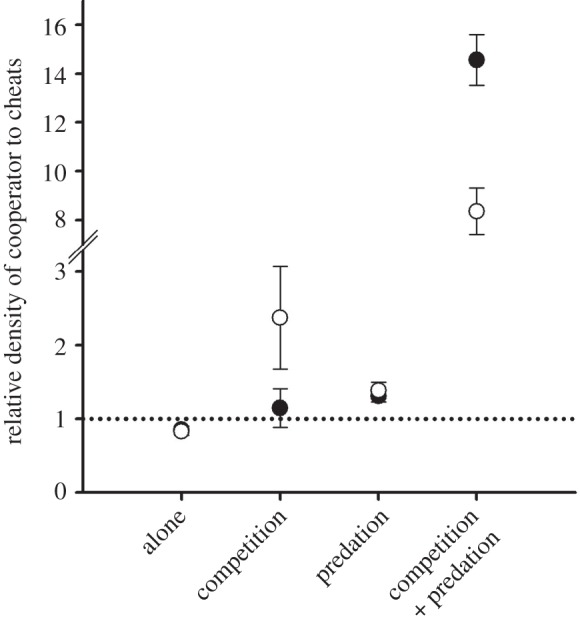
The relative density of cooperator (PAO1) to *lasI* (filled circles) and *lasR* (open circles) cheats in different experimental treatments in the lowest resource concentration. Values above the reference line denote relatively higher cooperator fitness and values below the line relatively higher cheat fitness. Bars show ±1 s.e.m.

**Figure 2.**
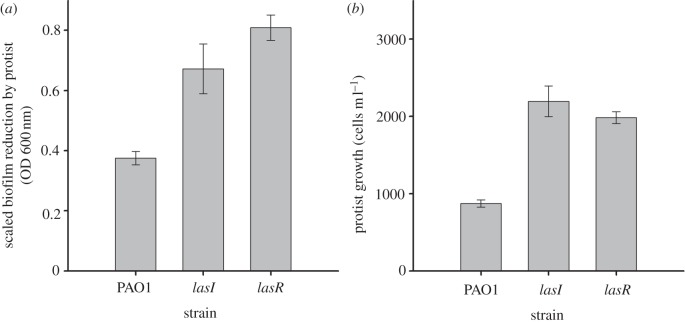
Bacterial traits measured in monocultures in the lowest resource concentration. (*a*) Protist ability to reduce cooperator and cheat biofilm. (*b*) Protist growth on cooperator and cheat strains. Bars show ±1 s.e.m.

All strains reached lower densities in the presence of competitor versus when grown alone (*F*_1,30_ = 72.8, *p* < 0.001, electronic supplementary material, figure S2), this effect being asymmetrical in favour of cooperator probably owing to difference in growth rate, interference competition or ability to use private goods. Similarly, predation decreased the densities of all strains both in the absence (*F*_1,12_ = 384, *p* < 0.001; electronic supplementary material, figure S2) and presence of competition (*F*_1,12_ = 842, *p* < 0.001; electronic supplementary material, figure S2). While both predation and competition reduced relatively more the frequency of cheats (predation × strain: *F*_2,30_ = 9.6, *p* = 0.001; competition × strain: *F*_2,30_ = 10.9, *p* < 0.001; figure 1; electronic supplementary material, S2), their interactive effects led to the greatest reduction in cheat frequency (predation × competition × strain: *F*_2,30_ = 4.8, *p* = 0.015, figure 1; electronic supplementary material, S2). In other words, cheats were unable to exploit the resistance mechanism of cooperators. The mechanism of resistance is not known for certain, but biofilms, which are known to confer resistance to protist predation, were more vulnerable to predation in cheat populations, (*F*_2,12_ = 16, *p* = 0.001; figure 2*a*) and this led to higher protist yield (*F*_2,12_ = 4.8, *p* = 0.02; figure 2*b*). Moreover, cooperators formed larger and denser cell aggregations in the liquid phase of the culture medium compared with both *lasI* and *lasR* cheats (see electronic supplementary material, figure S3).

Surprisingly, the increased resistance associated with an intact QS system appears to be independent of social aspects of QS itself: the presence of cooperator did not increase the growth of the signal-negative cheat (*F*_1,10_ = 1, *p* = 0.34, figure 1; electronic supplementary material, S2).

## 4. Discussion

Here, we show that protist predation provides a large fitness benefit to an intact QS system, which ultimately controls a range of social and non-social traits in the opportunistic pathogen bacterium *P. aeruginosa*. In contrast with the situation for many other QS-regulated traits, such as the production of proteases and toxins [7], here cheats were unable to gain a fitness advantage from the presence of cooperators; indeed, the negative impact of predation on cheats was increased in the presence of cooperators, presumably because of increased resource competition. Moreover, the presence of wild-type bacteria did not enhance the growth of the signal-defective *lasI* mutant. This suggests that QS signalling resulted in the expression of increased individual resistance (non-social resistance), and/or that resistance was social, but cheats were unable to exploit this social resistance.

How could such exclusivity of QS signalling, and potentially of public goods production, occur? The most likely explanation is the tendency of *P. aeruginosa* cells to form aggregations, either on surfaces (biofilms) or in the liquid phase of the culture (flocs). Even though exogenous signal did not affect the growth of signal-defective (*lasI*) cheats in the liquid phase of the microcosms, it is known that signalling occurs in biofilms [13], and concentration of signal in a biofilm may be sufficient for QS, even when concentrations in the media are not [5]. Mutations in QS genes reduce biofilm strength [14,15], and while cheats can invade biofilms [13], growth within an aggregation will, by definition, spatially structure the population, increasing the probability of cooperators being spatially associated with other cooperators. Crucially, being in a large cell aggregation has been shown to increase resistance against predation by ciliate protists [16,17]. We found here that cheat biofilms were more vulnerable to predation by protists yielding higher protist cell numbers, whereas cooperators formed larger and denser cell aggregations in the liquid phase of the culture medium. We therefore suggest that wild-type bacteria were more resistant because they could produce and respond to QS signals in biofilms, resulting in larger and stronger cell aggregations. Cheats were presumably unable to exploit these aggregations because of population structure: biofilm growth mode could exclusively favour cooperators by increasing relatedness [18]. Alternatively, biofilms containing both the wild-type and cheat could have been less resistant, and thus more readily consumed by protists [13]. In the case of signal-blind (*lasR*) mutants, it is also possible that their inability to QS even in the presence of wild-type bacteria made them individually more vulnerable to predation even when associated with an aggregation. Unfortunately, directly assessing the importance of cell aggregations to resistance is extremely difficult: *P. aeruginosa* forms aggregations even in constantly disturbed environments (shaken tubes [18]) making it hard to find a biofilm-free control environment.

In summary, our results demonstrate that trophic interactions, for instance predation, can affect the evolution of social interactions within species, potentially affecting the evolution of QS-signalling-regulated *P. aeruginosa* virulence in aquatic environmental reservoirs poor in nutrients.
